# Children and young people’s participation in decision-making within healthcare organisations in New Zealand: An integrative review

**DOI:** 10.1177/13674935231153430

**Published:** 2023-02-21

**Authors:** Mandie Foster, Julie Blamires, Chris Moir, Virginia Jones, Jagamaya Shrestha-Ranjit, Brie Fenton, Annette Dickinson

**Affiliations:** 1 School of Clinical Sciences, 1410 Auckland University of Technology, Auckland, New Zealand; 2 School of Nursing and Midwifery, Edith Cowan University, Perth, WA, Australia; 3 Child and Youth Health Research Centre, 1410 AUT, Auckland, New Zealand; 4 Centre for Postgraduate Nursing Studies, 2494 University of Otago, Christchurch, New Zealand

**Keywords:** children’s participation, children’s rights, child advocacy, determination of healthcare needs, healthcare systems

## Abstract

There is a paucity of literature on children and young people’s participation in decision-making within healthcare organisations in New Zealand. This integrative review examined child self-reported peer-reviewed manuscripts and published guidelines, policy, reviews, expert opinion and legislation to explore how New Zealand children and young people participate in discussions and decision-making processes within healthcare settings and what are barriers and benefits to such participation. Four child self-reported peer-reviewed manuscripts and twelve expert opinion documents were retrieved from four electronic databases including academic, government and institutional websites. Inductive content thematic analysis generated one theme (a discourse in children and young people’s participation within healthcare settings), four sub-themes, 11 categories, 93 codes and 202 findings. It is evident within this review that there is a discourse between what expert opinion are stating is required to promote children and young people’s participation in discussions and decision-making processes within healthcare settings and what is occurring in practice. Despite literature reporting on how children and young people’s participation and voice were essential for healthcare provision, there was sparse literature published on children and young people’s participation in discussions and decision-making processes in healthcare delivery in New Zealand.

## Introduction

There are more than 2.3 billion children under 18 years of age, 1.2 billion young people from 15 to 24 years of age and around six million under the age of 5 years in the world with nearly two billion living in a developing country (United Nations International Children's Emergency Fund, 2022). As defined by the United Nations and United Nations Convention on the Rights of the Child (UNCRC), a child is aged between 0 and 18 years and a young person is aged between 15 and 24 years (Save the Children, n.d, United Nations, n.d). In New Zealand (NZ) there are 1.1 million children and young people (CYP) under 18 years of age, representing 23% of the total population, with most CYP residing in Auckland (389,00), Wellington (94,200) and Christchurch (81,800) (Stats New Zealand, 2022). The greatest number of CYP are of European ethnicity (71%), followed by Māori (26%) and Pacific (14%); however, Māori and Pacific children are more likely to require healthcare services for conditions associated with deprivation (Children’s Convention Monitoring Group, 2018).

The NZ Government ratified the UNCRC in 1993 which tenants are to promote, respect, protect, and fulfil rights of all CYP in law, policies and practices. United Nations Convention on the Rights of the Child is ratified in 196 countries globally making it the most widely ratified human rights treaty in the world (Save the Children, n.d). United Nations Convention on the Rights of the Child not only seeks to protect CYP in all areas of society, but also takes a rights-based approach to CYP participating in sharing their views on things that are important to them. Article 12 and 13 calls for CYP’s participation in decision-making related to policy or service delivery, in a manner appropriate to their age and development (United Nations Human Rights Office of the High Commissioner, 1989). Key government agencies in NZ whose role is to ensure the UNCRC treaty is honoured include Office of the Children’s Commissioner, Human Rights Commission and Children’s Convention Monitoring Group (Stats New Zealand, 2022; Children’s Convention Monitoring Group, 2018). In 2016, the Children’s Convention Monitoring Group, which monitors NZ Government’s implementation of the UNCRC, put (Coyne et al., 2016; Foster and Shields, 2020). forward 105 recommendations to improve CYP’s rights in NZ, which included recommendations for increased CYP’s participation and engagement in co-design of healthcare strategies, shaping practice, setting policy, evaluating success, and designing services with providers, and governmental departments (Children’s Convention Monitoring Group, 2018).

In accordance with UNCRC, a child centred care (CCC) and/or child and family centred care approach used in synergy with the Best Interests of the Child Model (Kalverboer and Zijlstra, 2006) are methods used globally to honour CYP’s rights The child is at the forefront of care delivery, within both a CCC and a child and family centred care approach, placing them in the context of family and community. It is generally believed that both CCC and child and family centred care provide the most appropriate and aspirational care frameworks to ensure that CYP’s rights are upheld within healthcare settings (Kelly et al., 2012). The Best Interests of the Child Model is strongly aligned to UNCRC human rights treaty and includes 14 environmental conditions that shapes a CYP’s development (Kalverboer and Zijlstra, 2006). These environmental conditions have been reported in literature as important areas for healthcare professionals (HCPs) and organisations to consider in promoting CYP’s health, wellbeing, and voice, and is especially important to consider for CYP who are in care or excluded from having a voice due to protectionist positions (Bromley et al., 2020).

When addressing how CYP are involved in discussions and decision-making processes within healthcare settings ‘participation’ refers to the process of sharing decisions which affect one’s life, and life of the community in which one lives (Hart, 1992) (page. 5). As stated above, article 12 and 13 of UNCRC insists on participation of CYP in matters that concern them, and Hart’s ladder of participation provides a useful framework to help health practitioners and policy makers think about the design of CYP’s participation in these matters (Hart, 1992). While it is not always possible for CYP to operate on the highest rung of the ladder it should be a goal for HCPs to enable CYP to participate at the highest level possible. One of the paths towards the higher rungs of the ladder are co-design and participatory research which are important methodological approaches for HCPs, organisations and researchers to consider (Jones et al., 2020; King et al., 2022). Participatory research includes active involvement of CYP and promotes independence, and inclusion; ensures service reflects their needs and wishes, and therefore adds value to health service planning, and enhances a better quality of service (Jones et al., 2020). Co-design is a process that uses creative participatory methods to bring professional experience alongside CYP with lived experience that creates conditions for genuine partnership, inclusion, and meaningful participation with shared knowledge and power (King et al., 2022). In NZ the current Health Strategy has greater focus on engaging CYP in care to better understand their health needs (Minister of Health, 2016). The authors of this review propose that despite expert opinion, legislative, and policy documents stating CYP’s participation and voice is essential for healthcare provision, published evidence of CYP’s participation in healthcare delivery in NZ will be limited. This review will fill a gap in the literature on how HCPs, organisations and key stakeholders in NZ can involve CYP in shared decision-making that is responsive, meaningful and effective. It will help to build new understandings about barriers and facilitators for CYP participating in discussions and decision-making processes within healthcare settings to inform practice, theory and policy.

Aim: To explore how NZ CYP participate in discussions and decision-making processes within healthcare settings and what are the barriers and benefits to such participation.

## Method

Design: The method used to conduct this review was that of an integrative review as described by Toronto and Remington (2020). This allowed the authors to take a systematic approach while at the same time incorporating a diverse range of literature (Toronto and Remington, 2020).

Search Method: Four electronic databases (Cumulated Index to Nursing and Allied Health Literature, Elton B. Stephens Company, Scopus, and Psychological Information) including academic, government and institutional websites were searched to access peer-reviewed literature, guidelines, policy and legislation documents. Key search words were (1) NZ or New Zealand or Aotearoa, AND (2) children or adolescents or youth or child or teenager or pediatric or paediatric or kids, AND (3) participation or engagement or involvement OR decision making or decision-making process, AND (4) healthcare organisations or hospitals OR healthcare or health services or hospital or health facilities. Academic, government and institutional websites searched are represented in Table 1.

**Table 1.** Websites searched.

Websites	Child and Youth Wellbeing, CCS Disability Organisation New Zealand, Cure Kids, Department of the Prime Minister and Cabinet, Health Navigator New Zealand, Four Main Tertiary Hospitals within New Zealand, Kids Health, Medical Council of New Zealand, Ministry of Health, Ministry of Social Development, New Zealand Commissioner for Children, New Zealand Health and Disability Commissioner, New Zealand Human Rights Commission, New Zealand Nursing Organisation, Nursing Council of New Zealand, Paediatric Society of New Zealand, UNICEF New Zealand, United Nations Convention on the Rights of the Child, United Nations Convention on the Rights of Children Monitoring Group, Five University repositories within New Zealand, World Health Organisation.

### Inclusion criteria

Any published child self-reported peer-reviewed manuscript and published manuscript, guideline, strategy, expert opinion or policy document on CYP’s participation in discussions in decision-making processes within healthcare organisations in NZ from 1998–2022.

### Exclusion criteria

Published manuscript, guideline, strategy or policy document that did not meet inclusion criteria.

### Data extraction and synthesis

Peer-reviewed manuscripts, expert opinion and policy documents were analysed iteratively through inductive thematic content analysis (Boyatzis, 1998). Researchers critically reflected upon any pre-conceived assumptions they had at the beginning of the study to limit any potential bias. Data on phenomenon of the research question were underlined (findings), coded and grouped into smaller or larger categories, and themes based on similarity of meaning by two researchers. Researchers moved between the data and reviewed codes, categories and themes multiple times in a repetitive cyclic process iteratively until no new themes or categories were evident, and the research team felt the themes portrayed meaning and significance of the text (Boyatzis, 1998). If there were any disagreement between codes, categories and themes, further discussions were held with the research team until a consensus was reached (Boyatzis, 1998).

### Critical appraisal

Joanna Briggs Institute critical appraisal tools were used by two independent appraisers to assess overall quality of peer-reviewed and expert opinion manuscripts/documents, that were further discussed with a third reviewer (Joanna Briggs Institute, 2022). If there were any disagreement between scores these were then discussed with the research team until a consensus was agreed. No manuscripts or documents were excluded based on a low critical appraisal score.

## Results

### Search outcome

**Figure 1.**
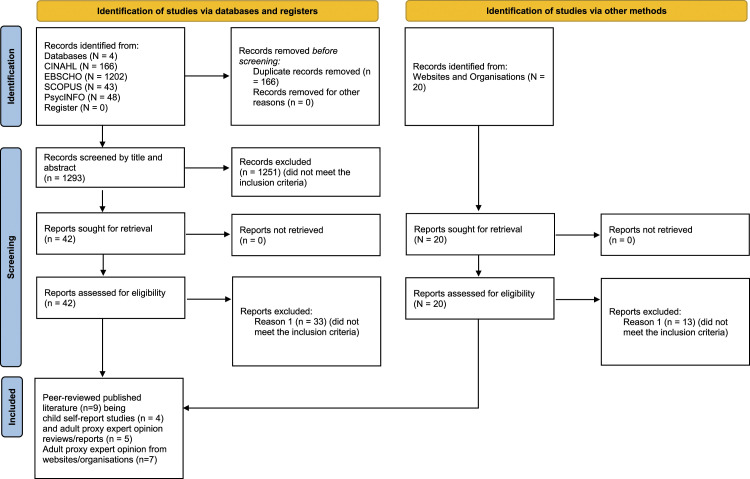
Flow diagram for integrative review.

Preferred Reporting Items for Systematic Reviews and Meta-Analysis (PRISMA) flowchart (Page et al., 2021) was used to explain the process of literature selection for this integrative review as indicated in Figure 1.

Four peer-reviewed manuscripts on CYP’s self-reported experiences on participation in discussions in decision-making processes within healthcare organisations in NZ were identified from databases (Gibson and Nelson, 2009; Gibson et al., 2016; Parbhu et al., 2019; Teevale et al., 2013) (Table 2). Twelve pieces of published evidence on adults perceptions/recommendations on CYP’s participation in discussions in decision-making processes within healthcare organisations in NZ were identified from databases, academic, government and institutional websites (Conder et al., 2016; Dickinson et al., 2014; Doell and Clendon, 2018; Human Rights Commission, 2010; McLean, 2000; Ministry of Health, 1998; Ministry of Social Development, 2003; The Paediatric Society of New Zealand, 2018; Eden-Mann, 2022; Van Rooyen et al., 2015; Wynd, 2015; Provoost, 2018). This included three reviews and nine expert opinion documents (Table 2). Critical appraisal scores ranged from four to 10 for CYP’s self-report manuscripts and from five to six for expert opinion documents (Table 2).

**Table 2.** Child self-report manuscripts and New Zealand child consumer policy documents – guidelines - reviews.

Child self-report manuscripts						
Author, date, country	Design type	Participants	Data collection	Results	Recommendations	JBI appraisal score
Gibson C and Nelson K (2009), New Zealand	Quantitative descriptive (conjoint analysis)	Twenty-nine young people, 19 (65%) were male 83% were of New Zealand European ethnicity and the mean age was 15.5 years with an age range 12 to 22 years	Conjoint analysis requires the sorting of options, in this instance cards describing service attributes, into most preferred/important to least preferred/important scenarios and included four stages	Importance of each attribute: Location of inpatient stay (29%), use of cell phone (28%), kitchen facilities (21%), bathroom facilities (11%) and recreation room (10%). The young people did not just talk of problems, but also of solutions	Adolescents have firm views and should always be consulted. Further research needs to include questions relating to having parents, siblings, friends stay, privacy and educational needs	Quantitative 4/10
Gibson, Cartwright, Campbell and Seymour (2016)	Qualitative study	Sixty-three young people, age range of 13–18 years, average age of 16.44 women, 18 young men and one person identifying as gender fluid	Participants were interviewed in an open-ended interview about the psychological service they had accessed. Data was transcribed then coded separately. Codes identified were used to develop into themes that reflected areas of priority	Participants prioritised their ability to retain control, the importance of privacy with no parental involvement. The importance of having a good relationship with their counsellor and being able to talk and be listened to. While expressing desire for accessible and flexible psychological services	Adolescents may have common priorities and concerns that affect engagement with services. Privacy and no parental involvement were important	Qualitative 10/10
Parbhu, Reay, Landhuis and Water (2019)	Qualitative and quantitative mixed methods	32 children, 45 parents and 12 nurses. 60% were NZ European decent. Age of children 5–18. Gender nor reason for admission was not collected	Children were given the use of an existing IV pole as well as the sprout IV pole. A crossover design was used where half the children were given the existing IV pole and switched to the sprout pole and vice versa. Parents filled out a questionnaire independently and research assistants helped the children to complete theirs to avoid parental input. Nurses were emailed separately	Quantitative findings: Safety was rated the most important followed by mobility and functionality with aesthetics being the least important. Nurses expressed greater importance for mobility and functionality to be more important than children and parents Qualitative findings: Importance of aesthetics in contributing to medical supplies being perceived as child-friendly	Design is important to children, therefore engaging them in the design of medical products and their perspectives is essential. Gives them greater control over preferences and autonomy. Researchers to establish good relationships and communication with hospital gatekeepers to help navigate inclusion of children	Qualitative 6/10 Quantitative 9/10
Teevale, Denny, Percival and Fleming (2013)	Quantitative descriptive	5975 secondary school students were included. 1178 were pacific youth aged from 13 to 18	Data looked at which students had accessed/forgone healthcare services in the last 12 months, as well as difficulty and reasons for not accessing healthcare	Results of study add to previous research which shows that pacific people experience barriers in access and use of services across NZ’s health and disability system. Greater communication between healthcare professionals, cultural and linguistic competencies to the patient, results in greater quality of care and satisfaction for the patient. Research elicits young people’s perspectives on how to counter perceived discrimination	Good access and utilisation of primary care services is an important factor in preventable health for Pacific New Zealanders	Quantitative 4/10

New Zealand child consumer policy documents – Guidelines - reviews				
Author, date	Type	Key components	Recommendations	JBI appraisal score
Conder, Schmidt and Mirfin-Veitch (2016)	Literature review	New Zealand’s response to international directives, are children with disabilities’ voices are different to all children’s voices, research methods for children with disability, interviews as a research method with children with disabilities and ethical considerations	15 recommendations that covered communication, support, clear goals, a willingness to listen, ambiguity, consideration of socio-cultural, age and disability specific variables, age-appropriate research methodologies (drawing, talking mats, photography, cue cards, pictures, tape recording, questionnaires with adaptations, if necessary, dolls or similar toys, story-telling, drama, digital and other media, music, and observation) and devices, feedback to children and resources	Expert opinion 6/6
Dickinson, Wrapson and Water (2014)	Literature review	Widespread acceptance in that children’s opinions should be sought in their own affairs. Children’s views often obtained by proxy. Exploration of MOH and HQSC to discover what input children already have. Findings show that children’s views are gathered through proxy via MOH, HQSC and DHB’s	Any new approach to gathering patients’ experiences/opinions should include children of all ages to avoid disengaging younger children. Culturally appropriate and relevant methods should be developed	Expert opinion 6/6
Doell and Clendon (2018)	Literature review - expert opinion	Key policies and strategic direction for enhancing children’s rights to be involved in development of services. Review of international literature. Recommendations for structured information services, establishing partnership models and capability building	Consultations with SLP1 will identify systems, professional competencies and interpersonal skills that support information sharing, service coordination, holistic practice and partnership approaches. Time and resources are essential to build relationships	Expert opinion 6/6
Human Rights Commission (2010)	Expert opinion	Talks about UN rights of children and what is currently in place and what needs to be improved	Increase avenues for children to participate in and to be listened to too	Expert opinion 6/6
McLean (2000)	Expert opinion	Looks at the status of Gillick in NZ, what the Gillick principle is and proposal for furthering Gillick	Implementing the Gillick principle to assess competence of minors to allow them the right to decision-making and input in healthcare while also allowing them the right to refuse treatment	Expert opinion 6/6
Ministry of Health (1998)	Expert opinion	Discusses consent, children’s rights communication with children and their families. Legal aspects that cover children in terms of healthcare and consent	How to ensure consent is a part of healthcare practice. Implementation of the Gillick principle in a NZ healthcare setting	Expert opinion 6/6
Ministry of Social Development (2003)	Expert opinion	How and why to involve children in participation and decision-making	Ethical considerations, addressing practical issues, how to work with different groups of children and the importance of gaining feedback	Expert opinion 5/5
The Paediatric society New Zealand (2018)	Expert opinion	Looks at the rights of children in healthcare settings	11 recommendations, 1 per each right of the child on how to implement each right when treating a child/youth	Expert opinion 6/6
Provoost (2018)	Expert opinion	Shows themes and values that children uphold regarding wellbeing	Listen to children to keep key elements at heart of frameworks and models. Shift thinking from government priorities to children’s priorities	Expert opinion 5/5
Eden-Mann (2022)	Expert opinion	The rights of children, the basics of shared decision-making and potential barriers. Guidelines, strategies and tools for working with young disabled people	Use different forms of communication rather than just verbal. Use drama or dance activities so that children understand procedures/treatments. Observing and interacting with the child in a variety of settings, consulting with people surrounding young person on unique communication methods	Expert opinion 5/5
Van Rooyen, Water, Rasmussen & Diesfeld (2015)	Expert opinion	Discusses what competency is, the importance of recognising competent children and what is known about NZ health law, policy and child competency	Further action from both academic and governmental areas to address issues faced in determining the competence of a child. Exploration of key attributes of child competence is required to assist health professionals in identification of competent children which in turn should inform more clear and practical policies on the subject	Expert opinion 5/5
Wynd (2015)	Expert opinion	Key disability issues in NZ. How disabled children are more likely to experience and be impacted by poverty. What current services MOE, MOH and MSD are doing to support disabled children and their families. Looks at the need for financial support, diagnoses’, difficulty accessing services, lack of knowledge for parents	Government collect and disseminate better quality publicly available data of the number, location and socioeconomic of disabled children. Acknowledge the role of special education needs coordinators in schools and accordingly funding resources. Funding and allocation of services be renewed in partnership to identify shortfalls. Office of disability issues investigate ways to ensure government agencies and staff recognise and consider the needs of disabled children and their families. Eligibility criteria for SLP2 be revised for parents. MSD to investigate barriers and access to CDA.	Expert opinion 5/5

CDA, child disability allowance; DHB, district health board; HQSC, health quality and safety commission; IV, intravenous; MOE, ministry of education; MOH, ministry of health; MSD, ministry of social development; NZ, New Zealand; SLP1, speech language pathologist; SLP2, supported living payment; SPSS, statistical package for the social sciences; UN, united Nations.

An inductive analysis of the findings generated one theme (a discourse in CYP’s participation within healthcare settings), four sub-themes (ethical considerations, service delivery, a child’s understanding, best interest of the child), 11 categories (respect, agency, research, competency, organisation, desires, communication, information, child’s perspective, adult perspective, participation), and included 93 codes and 202 findings (Table 3).

**Table 3.** Themes: Child self-report manuscripts and New Zealand child consumer policy, expert opinion and review documents.

Theme	Sub-theme	Category (number of codes)	Exemplar and (representative code)
A discourse in children and young people’s participation within healthcare settings	Ethical considerations (37 codes, 82 findings)	Respect (13)	Recognising competent children not only supports ethical arguments regarding respect for children’s rights and their personhood; it has other more tangible benefits to both the child and healthcare services (children’s rights) It is important that you have the attitude ‘I can learn from children’ (respect children’s views)
		Agency (5)	Children generally recognise that there will be limitations in regard to their agency and they value support in decision-making from people with whom they have a meaningful relationship (limitations in regards to agency) The use of proxy data is based on an assumption that children are dependent, incompetent, lack rationality and that somehow their views are less valid than those of adults (place in society)
		Research (13)	When interviewing allow for more than one meeting to assist with building rapport, be guided by non-verbal as well as verbal communication, be familiar with and use questioning strategies that encourage the child to accurately share their opinion and knowledge, and allow the child time to answer (child-friendly data collection methods) Different components of the health setting appear to be prominent at different ages, demonstrating the importance of including the views of both younger and older children (representation of age brackets)
		Competency (6)	The child’s or young person’s capacity is situation-specific and will depend on his or her experience in the same or similar situations, rather than on his or her age or intelligence (child’s capacity is fluidic) Health service providers also have an obligation to give due weight to this opinion in accordance with the competence of the child or young person (child’s competence)
	Service delivery (25 codes and 51 findings)	Organisation (7)	The 2012/13 children’s survey contained seven questions about usage of public hospital services, but none concerned perceptions of the care received (care received) Health service providers have an obligation to respect the child’s or young person’s right to express their view in all matters affecting them (service delivery)
		Desires (18)	She asks about my life and how I’m doing, not just about my problems. When she asks me about my life I know she’s genuinely asking me because she’s interested (building a relationship) It was helpful when they would sit down with me and say you get to choose the direction in your life (choices)
	A Child’s understanding (9 codes and 17 findings)	Communication (4)	Health service providers should attempt to understand and make sense of children’s and young people’s communications, rather than assume that they do not make sense (child communication) It is crucial that health professionals talk directly to children and young people, as well as to their families/whānau, even if the child or young person may seem unable to comprehend (direct communication)
		Information (5)	Children and young people have a right to information that they can understand about their health and healthcare. This includes information about the choice of health care services available (age-appropriate information) It is the responsibility of health professionals to impart information in a way that supports children developing health literacy as this influences a child’s ability to process and understand their conditions and options (children’s health literacy)
	Best interests of the child (22 codes and 52 findings)	Child’s perspective (7)	Special attention and some creativity are often necessary to ensure that children have the freedom to seek, receive and impart information and ideas, not only orally but also through other means of the child’s or young person’s choice, such as play and art (creative feedback) To be honest I just like to talk. I Just like having someone listen to me (someone listening to me)
		Adult perspective (4)	Historically, both in New Zealand and elsewhere, information about children’s views has been obtained via proxy accounts given by adults – primarily parents or carers – or has relied on the stories of older adolescents (parent proxy) However, children and adults have different experiences, understandings and perspectives and there may be differences in what is perceived as important in a healthcare setting. Consequently, children’s views and those of adults may not always concur (parent child discourse)
		Participation (11)	I wasn’t keen on having that meeting that day because I don’t want to be there and because my parents were there and I was kind of put off...So it got kind of hard during that first session because I didn’t say anything...I wouldn’t say anything (not wanting to be there) Despite recognition of the need to include children in the development and improvement of healthcare systems, there is still a lack of opportunities in New Zealand for participation (lack of opportunity)

### A Discourse in Children and Young People’s Participation within Healthcare Settings

A discourse in CYP’s participation within healthcare settings in NZ included the sub-themes ethical considerations, service delivery, a child’s understanding, and what was perceived as in the best interest of CYP. Despite literature reporting on how CYP’s participation and voice were essential for healthcare provision, there was sparse literature published on CYP’s participation in discussions and decision-making processes in healthcare delivery in NZ.

### Ethical considerations

Ethical considerations are underpinned by four categories (respect, agency, research, competency), and included 37 codes and 82 findings. The literature highlighted the importance of respecting CYP’s views by ensuring adult conversations with CYP are meaningful with adults displaying a receptive attitude to learn from CYP (Ministry of Social Development, 2003). Conversations should ensure the course of action, benefits, outcomes or harm of participation is in the child’s best interest, capacity and rights as stated by CYP (The Paediatric Society of New Zealand, 2018; Van Rooyen et al., 2015). Concepts identified as important in promoting respectful participation in decision-making discussions include providing a safe environment and honouring CYP’s privacy (Gibson et al., 2016; Ministry of Social Development, 2003; Parbhu et al., 2019), promoting and empowering CYP’s autonomy to participate (Conder et al., 2016; Ministry of Health, 1998; The Paediatric Society of New Zealand, 2018), and avoiding tokenism (Dickinson et al., 2014; Ministry of Social Development, 2003).

Children and young people’s agency refers to acknowledging CYP as competent capable individuals that can contribute to their own care and wellbeing, and dismissing the historical paternalistic viewpoint where CYP are viewed as vulnerable, dependent, incompetent, irrational, and that somehow their views are less valid than those of adults (Dickinson et al., 2014; Human Rights Commission, 2010). While, very young children can be valuable competent informants in regard to their care and to society as a whole (Dickinson et al., 2014), children themselves may be aware of their own limitations to their agency and value support from people they trust (Conder et al., 2016).

As highlighted in the Humans Rights Commission (2010) and UNCRC article 12 (1), CYP should be provided with correct information, options and opportunities to participate in healthcare delivery and research (Eden-Mann, 2022; Ministry of Health, 1998). Children and young people’s participation and decision-making in research needs to be creative, appropriate, realistic, utilising child-friendly designs/methods, where sampling ensures a wide representation of age brackets (Conder et al., 2016; Dickinson et al., 2014; Eden-Mann, 2022). Some of the reported effective child-friendly data collection methods include ‘drawing, talking mats, photography, cue cards, pictures, tape recording, questionnaires with adaptations if necessary, dolls or similar toys, story-telling, drama, digital and other media, music, and observation’ (Conder et al., 2016) (p. 23).

Further effective child-friendly research methods include providing CYP with more than one meeting to assist relationship building, being aware of non-verbal and verbal communication, and allowing CYP time to answer questions (Conder et al., 2016; Ministry of Social Development, 2003). All research activities undertaken with CYP in NZ need to be underpinned by a maturity-based approach that supports the applicability of the Gillick case with clear goals that CYP can understand, including opportunities for CYP to provide real time feedback to facilitate change (Conder et al., 2016; Dickinson et al., 2014; McLean, 2000; Van Rooyen et al., 2015).

Children and young people’s competency to consent to participate in decision-making needs to be undertaken within an age-appropriate CCC approach taking into consideration CYP’s developmental level, age, illness typology, experiential knowledge, state of mind, capacity and an awareness that CYP’s competency to consent is in a constant state of movement (Dickinson et al., 2014; McLean, 2000; Ministry of Health, 1998; The Paediatric Society of New Zealand, 2018; Van Rooyen et al., 2015). Similarly, CYP living with a disability should not be seen as a homogenous group and require special consideration in relation to their socio-cultural, disability specific variables (Conder et al., 2016; Eden-Mann, 2022; Wynd, 2015). Children and young people living with a disability need to be provided with the same respect for personal dignity, autonomy, self-determination and opportunities to participate in decision-making as any other person (Eden-Mann, 2022; Human Rights Commission, 2010; Ministry of Health, 1998; Ministry of Social Development, 2003). It has been reported that recognising CYP as competent decision makers has improved treatment adherence, clinical effectiveness, disease prevention and delivery of health services to CYP (Van Rooyen et al., 2015). This recognition ensures CYP learn to advocate and take responsibility for their own health, their personal development, and participation in society rather than having instantaneous responsibility at the age of sixteen (Van Rooyen et al., 2015).

### Service delivery

Service delivery is underpinned by three categories (organisations, desires, models of care), 25 codes and 51 findings. The literature reported healthcare delivery needs to be operationalised through a CCC approach with CYP, families/whānau and HCPs collectively collaborating to understand the CYP’s world within the context of family (Dickinson et al., 2014; The Paediatric Society of New Zealand, 2018). Healthcare services provided by organisations need to be better coordinated and accessible to CYP and provide equality of inputs and outcomes, without discrimination based on ethnicity, race, economic status, religion, gender, age, sexual orientation, disability, illness, appearance, language or culture (Ministry of Social Development, 2003; The Paediatric Society of New Zealand, 2018; Wynd, 2015). Although, these standards of equitable care are acknowledged as integral to good care, evidence indicates these goals are not always actualised, for example, Pacific adolescents reported significant barriers and inequity in accessing healthcare services for an injury, smoking program, asthma, pregnancy advice, dental care, and alcohol or drug use in comparison to their NZ European peers (Teevale et al., 2013).

When young people were asked to participate in discussions on what was important to them in hospital, some young people wanted to have a sibling or school friend stay overnight, have somewhere to watch television where they could socialise, and wanted staff to take time to get to know them (Gibson et al., 2016; Gibson and Nelson, 2009; Parbhu et al., 2019). In the Parbhu et al. (2019) study, children’s input into the design of a new intravenous pole was essential as children were able to let researchers know what was important, such as the pole design was friendly, strong, colourful, modern, with better functionality that provided them with greater confidence, independence, freedom and ability to play.

Altering the hospital environment and delivery of care based on CYP’s input is reported as gold standard yet, co-designing projects with CYP or research led by CYP needs to ensure CYP have appropriate resources, support and agreements in place with CYP’s involvement acknowledged as paramount to ensure the design and delivery of health services is child-centred (Conder et al., 2016; Dickinson et al., 2014; Ministry of Health, 1998). The literature further states the importance in making sure CYP find the experience of participation enjoyable and rewarding (Ministry of Social Development, 2003).

### A child’s understanding

A child’s understanding is underpinned by two categories (communication, information), nine codes and 17 findings. The evidence reported HCPs should consider using specific devices in communicating with CYP that CYP are familiar with and be cognisant of CYP’s verbal and non-verbal cues of communication (Conder et al., 2016; The Paediatric Society of New Zealand, 2018). Communication should be directed at CYP in collaboration with family/whānau with attempts made to fully understand CYP’s voices (Conder et al., 2016; The Paediatric Society of New Zealand, 2018).

The literature states information should be provided to CYP in an age-appropriate supportive manner as CYP have a right to information they can understand, including information about health literacy, and choices of healthcare services available to them (Van Rooyen et al., 2015). Some CYP may not want to receive information or participate in decision-making discussions, so information sharing should always be based on what CYP state they require (Ministry of Health, 1998; The Paediatric Society of New Zealand, 2018). It was reported that CYP need to be aware of their role in discussions that affect them and be provided with enough time to comprehend and digest what is being asked, as CYP’s ability to understand, changes and modifies with their experiences and social context (Ministry of Social Development, 2003; Van Rooyen et al., 2015).

### Best interest of the child

Best interest of the child is underpinned by three categories (CYP’s perspective, adult perspective, participation), 22 codes and 52 findings. Children and young people need to know that they have an important voice in discussions concerning them, and further be provided with the opportunity to see the outcomes of their involvement in policy and practice (Conder et al., 2016; The Paediatric Society of New Zealand, 2018; Van Rooyen et al., 2015).

Including CYP in shared decision-making is reported as being a positive means of empowering young people to contribute to processes and systems that affect their health and wellbeing (Dickinson et al., 2014). Historically, in NZ CYP’s views have been obtained via proxy accounts given by adults (parents, caregivers, staff), which have left CYP voiceless (Dickinson et al., 2014; Eden-Mann, 2022; The Paediatric Society of New Zealand, 2018). For example, a survey undertaken on insights into CYP’s access to and experience of primary and secondary healthcare services was obtained from parents/caregivers who acted as proxies for their child (McLean, 2000). Considering CYP’s perceptions and understanding of healthcare experiences may differ from their parents; to evaluate, change or alter services that affect CYP, gaining the CYP’s viewpoint is paramount (Dickinson et al., 2014; Ministry of Social Development, 2003).

For CYP to participate in shared decision-making there needs to be more opportunities available for CYP to become involved in matters that are of direct interest to them (Dickinson et al., 2014). Some reported barriers include a lack of opportunity for CYP’s perspectives to be taken into account, limited extent of direct questioning into CYP’s experiences, a perception that CYP lack experience to participate, difficulties with communication, not valuing CYP’s views, a lack of resources and time, thinking that it’s inappropriate to involve CYP in decision-making, not knowing how to address safety and ethics issues, thinking that CYP don’t want to participate, language and culture, not knowing how to involve CYP or how to discuss issues with them, thinking that the processes are too complex and time-consuming, financial constraints, and a lack of interest (Eden-Mann, 2022; Ministry of Social Development, 2003; Teevale et al., 2013). Appropriate and effective shared decision-making is guided by several principles some of which are that adults acknowledge the importance of including CYP’s voices, there are realistic expectations, and clear goals in line with CYP’s capacity (Ministry of Social Development, 2003).

## Discussion

This integrative review identified how CYP participate in discussions and decision-making processes within NZ healthcare settings and what the barriers and benefits were for such participation. Although, key informing documents and the literature articulated the benefits of CYP’s participation in healthcare, it was challenging to find examples in the published literature on how this was actualised in practice (Ministry of Social Development, 2003). It is useful to consider Roger Hart’s Ladder of Participation with its eight levels of CYP participation based on the interactions of power between adults and CYP (Hart, 1992). In our review, CYP were participating in a way that Hart (1992) would describe as ‘tokenistic’, where the documents and studies were adult-led, and where CYP may have been consulted but with minimal opportunities for feedback. Although not directly related to healthcare or healthcare organisations an excellent example of CYP’s participation in practice is the work that was undertaken by the Department of the Prime Minister and Cabinet in partnership with Office of the Children’s Commissioner and Oranga Tamariki, which enabled CYP’s views and experiences to inform the development of Child and Youth Wellbeing (Department of the Prime Minister and Cabinet, 2019a, 2019b; Office of the Children's Commissioner and Oranga Tamariki, 2019). In this example, a large sample of CYP in NZ participated in an online survey and focus group interviews. Children and young people’s narratives directly impacted and influenced developed outcomes within the Child and Youth Wellbeing Strategy (Brown et al., 2020). Given interest and support for CYP’s participation in healthcare decision-making and the contrasting lack of evidence in child self-reported published literature, this review highlights a need for more research to determine effective methods for supporting CYP’s participation in decision-making within healthcare.

In this review, the most common barriers included adult’s perceptions of CYP’s agency, its value, and importance, communication issues specifically with younger children or children with disability, the concept of protection, lack of resources/support or tools to engage CYP in a developmentally appropriate way, CYP’s expertise based on experiential knowledge, child representativeness, organisational culture and ethical issues related to CYP’s informed voluntary consent/assent. Similar barriers have been reported in international literature where HCPs took on a protective role by intercepting CYP’s ability to participate and/or focused on parents’ needs and the practical and ethical challenges of including CYP into service delivery, with little focus on the possibilities of CYP’s participation and strategies which may be used to mitigate these barriers (Coyne and Harder, 2011; Kiili et al., 2021). Further reservations to include CYP in discussions and decision-making processes included the CYP’s age, their vulnerability, maturity, CYP being viewed as reliant on parental proxy, including the generational position where CYP historically have been viewed as minors, and not partners in care (Davies et al., 2019; Kiili et al., 2021). Unlike other countries such as the United Kingdom, in NZ, there are no standards or regulatory requirement for health providers to show evidence of CYP’s involvement as consumers of healthcare or an agency which monitors such input.

The benefits of CYP’s involvement reported in this review included CYP’s voice not only informing service delivery and care but further improving treatment adherence, clinical effectiveness, disease prevention, confidence, satisfaction, motivation, and providing CYP an opportunity to take responsibility for their own health and development. Similar benefits have been reported in international literature where increased motivation, situated understanding, trusting relationships, security and comfort, sharing of ideas, feeling valued and supported to be involved, being listened to, and receiving information were reported by CYP (Carlsson et al., 2021; Stålberg et al., 2019). Of interest, most of the reported benefits and barriers in this review were from expert opinion and not directly from NZ CYP, and as reported in international literature a child’s perception compared to adult proxy can be quite different (Söderbäck et al., 2011). Hence, it is vital to include CYP’s voices and acknowledge what CYP self-report is important and needed to inform research, care delivery, treatment, and practice as in line with principles of the UNCRC, CCC, and the Best Interests of the Child Model, Children’s Commissioner Act (2003) and Convention and Human Rights Act (1993) (Foster et al., 2022; Ministry of Justice, 2022; Parliamentary Counsel Office, 2020).

Despite recommendations from experts, to include CYP as co-researchers, using co-design methodologies, and friendly data collection techniques with clear consideration to ethical issues, and CYP’s understanding of their involvement, representation of CYP’s views were limited in this review. It was noted by Dickinson et al. (2014) that there remains a significant gap in CYP’s representation in research to inform care delivery and practice within NZ healthcare settings. If researchers are going to use a co-design approach with CYP, then they need to reflect on various participatory designs at a meta level including consideration of participatory action research and participatory workshops (Bowler et al., 2021), and be willing to take a more collaborative interactive and creative approach to research (Jones et al., 2020; King et al., 2022). But this requires a shift from the traditional way in which CYP’s experience have been researched and acknowledgement that such an approach represents gold standard for gaining CYP’s input into design and delivery of healthcare services (Coyne et al., 2016; Jones et al., 2020; King et al., 2022). Such an approach, which includes CYP’s involvement in the conception, design, collection of data, analysis, reporting, and evaluation will ensure generation of findings that are meaningful, culturally appropriate and informative to delivery of healthcare services to CYP in NZ. Facca et al. (2020) state there is a need to move away from conceptualising child’s voice to instead theorising ‘voice’. They suggest that researchers explicitly attend to the methodological approach for ‘voice’, and its influence on how data are generated, analysed and presented. This includes acknowledgement that a child’s voice is relational (constructed through interactions with others), has no authentic point of origin, so needs multiple interpretations, and is produced through intergenerational dialogue with people of all ages (Facca et al., 2020). This integrative review will inform the Office of the Children’s Commissioner, Human Rights Commission and Children’s Convention Monitoring Group on the present CYP participation initiatives within NZ, to direct future CYP participation initiatives, interventions, guidelines, legislation, practice and delivery of healthcare services to CYP in NZ.

### Limitations and strengths

The included manuscripts on young children’s voices were limited, with no published literature evident from children less than 5 years of age. The results may have been influenced by the large number of expert opinion documents and potential bias among the research team. In addition, international evidence was excluded as the authors explored literature solely undertaken within NZ. Finally, CYP were not consulted on the results of this integrative review, given the project commenced during the COVID-19 pandemic. Despite these limitations the procedure undertaken by the authors in this review was rigorous and followed a systematic process.

### Implications for Practice and Research

Listening to CYP, including them, and enabling them to be involved in healthcare will help CYP feel respected and has potential to positively impact future healthcare interactions. Implications if strategies are not put in place is that CYP’s voices on healthcare matters that concern them will continue to remain silent and/or be represented by adults. Further research is required on CYP’s and HCP’s perceptions on CYP’s agency as competent social actors who can participate and co-construct situations with others in healthcare delivery from an individual and situational lens. It would also be beneficial for future research to explore how to address or overcome the barriers identified in this review.

## Conclusion

The results of this review may help HCPs, policy makers and researchers to understand what influences CYP’s possibilities to participate in healthcare decisions. The barriers described are interconnected and therefore overcoming or addressing even one of these has potential to generate positive outcomes. The findings highlight the importance of supporting participation of CYP on multiple levels and in different situations to meet the requirements of the UNCRC. In essence including CYP’s participation in discussions and decision-making processes within healthcare settings requires a contextually embedded multi-tiered lens approach with careful attention to critical reasoning and situational knowledge.
